# Rate of Free Flap Failure and Return to the Operating Room in Lower Limb Reconstruction: A Systematic Review

**DOI:** 10.3390/jcm13154295

**Published:** 2024-07-23

**Authors:** Pietro Luciano Serra, Filippo Boriani, Umraz Khan, Matteo Atzeni, Andrea Figus

**Affiliations:** 1 Plastic Surgery Unit, Department of Medical, Surgical and Experimental Sciences, University of Sassari, Sassari University Hospital Trust, 07100 Sassari, Italy; 2 Plastic Surgery and Microsurgery Unit, Department of Surgical Sciences, Faculty of Medicine and Surgery, University Hospital “Duilio Casula”, University of Cagliari, 09124 Cagliari, Italy; borianifilippo@gmail.com (F.B.); teo.atzeni@gmail.com (M.A.); andreafigus@hotmail.com (A.F.); 3 Plastic and Reconstructive Surgery Department, North Bristol NHS Trust, Southmead Hospital, Southmead Road, London BS10 5NB, UK; umrazk5@gmail.com

**Keywords:** lower limb, reconstruction, free flap, failure rate, return to the operating room

## Abstract

**Background:** Soft tissue defects of the lower limbs pose significant challenges in reconstructive surgery, accounting for approximately 10% of all reconstructive free flaps performed. These reconstructions often encounter higher complication rates due to various factors such as inflammation, infection, impaired blood flow, and nerve injuries. **Methods:** A systematic review was conducted following PRISMA guidelines, reviewing literature from 2017 to 2024. Eligible studies included those on free flap reconstruction of lower limb defects in living human subjects, with more than three cases and reported rates of flap failure and return to the operating room. Systematic reviews and metanalysis were excluded. **Results:** A total of 17 studies comprising 5061 patients and 5133 free flap reconstructions were included. The most common defects were in the lower leg (52.19%) due to trauma (79.40%). The total flap necrosis rate was 7.78%, the partial necrosis rate was 9.15%, and the rate of return to the operating room for suspected vascular compromise was 13.79%. **Discussion:** Lower limb reconstruction presents challenges due to diverse etiologies and variable tissue requirements. Factors such as recipient vessel availability, flap selection, and multidisciplinary approaches influence outcomes. Muscle and fasciocutaneous flaps remain common choices, each with advantages and limitations. This systematic review underscores the importance of individualized treatment planning. **Conclusions:** Microsurgical reconstruction of lower limb defects demonstrates safety and reliability, with overall favorable outcomes. Flap selection should be tailored to specific patient needs and defect characteristics, emphasizing meticulous surgical techniques and multidisciplinary collaboration. This systematic review provides valuable insights into current standards and encourages adherence to best practices in lower limb reconstruction.

## 1. Introduction

Soft tissue defects of the lower limbs are common, constituting ~10% of all reconstructive free flaps performed by plastic surgeons according to the 2019 United Kingdom National Flap Registry [1].

Lower limb free flap reconstruction can be technically challenging and usually has higher rates of complications compared to other anatomic sites [2,3,4,5,6,7,8,9]. Numerous factors can complicate a given defect, such as inflammation, infection, impaired blood flow, lymphatic damage, unstable skin, and nerve and/or osseous injury [10,11,12]. These aspects must be balanced with the reconstructive priorities of providing stable, aesthetically pleasing coverage that fosters functional restoration and eventual union of osseous injuries and is accomplished with minimal donor-site morbidity [13]. Nevertheless, free tissue transfer has a vital role after traumatic injury or oncologic resection because of its ability to place healthy, vascularized tissue, obliterate dead space, and promote osseous union.

Historically, Godina’s paper [7] published in 1986 paved the way for free-flap reconstruction for defects in lower extremities. Muscle flaps were considered to be superior to fasciocutaneous flaps because of their capacity to fill dead space. Moreover, it was believed that their reliable vascular supply decreased microbial load and promoted bone union [14,15,16,17]. On the other hand, fasciocutaneous flaps avoid muscle sacrifice and may result in a better aesthetic contour with thin, pliable tissue [18,19]. However, many studies demonstrated the same reconstructive, functional and bone union outcomes between muscle and fasciocutaneous flaps [20,21,22,23,24].

When Koshima published his paper [25] in 1989, he marked the beginning of the perforator flaps era, and there was a paradigm shift in the reconstruction of the lower extremities [26,27,28]. Then, in 1994, Gottlieb and Krieger [29] described the “reconstructive elevator” where the simplest method is not always the best and plastic surgery should pursue a creative thought rather than a sequential one.

Nowadays, the heterogeneous nature of causes of defects in lower limb has precluded the determination that a specific flap type is the ideal choice for all defects. In light of this, flap choice should be tailored to meet the needs of each particular injury [30].

In fact, the progress in and high success rates of microsurgical free flaps allow large and composite tissue defects, regardless of their causes, to be covered using numerous different types of autologous tissue and expanding our reconstructive armamentarium.

The aim of this systematic review was to analyze the recent published literature regarding the rate of flap failure and return to the operating room in microsurgical reconstruction of the lower extremity. An analysis of the flaps, sites and principal causes of the defect has also been conducted.

## 2. Methods

This systematic review was conducted using the Preferred Reporting Items for Systematic Reviews and Meta-Analysis (PRISMA) guidelines [31]. The article has not been registered.

The literature was examined by three independent reviewers through the PubMed (MEDLINE), EMBASE and Scopus databases using the keywords “Free”, “Flap”, “Lower”, “Limb”, “Limbs”, “Extremity”, and “Reconstruction”, applying the Boolean operators “OR” and “AND”. The search involved articles published in the last 7 years (2017–2024).

Eligibility was determined using the following inclusion and exclusion criteria. Inclusion criteria included studies pertaining to free flap reconstruction of the lower limb for any cause, case series of more than three patients, studies where the rate of total and partial flap failure and rate of return to theatre were present, English language publications, and studies conducted on living human beings.

Exclusion criteria included single-series case reports, systematic reviews, metanalysis, upper extremity reconstructions such as toe-to-digit transfers, reconstructions with pedicled flaps, studies using additional therapies (WNP, specific drugs), studies in languages other than English, and studies on animals or cadavers.

Citations found through the database search were screened for eligibility first by title, then by the abstract, and finally by the full text.

Our primary outcome was to assess the rate of free flap total and partial necrosis and the rate of return to the operating room for suspected vascular compromise. Methodological quality was assessed using the MINORS criteria and level of evidence.

## 3. Results

**Figure 1:**
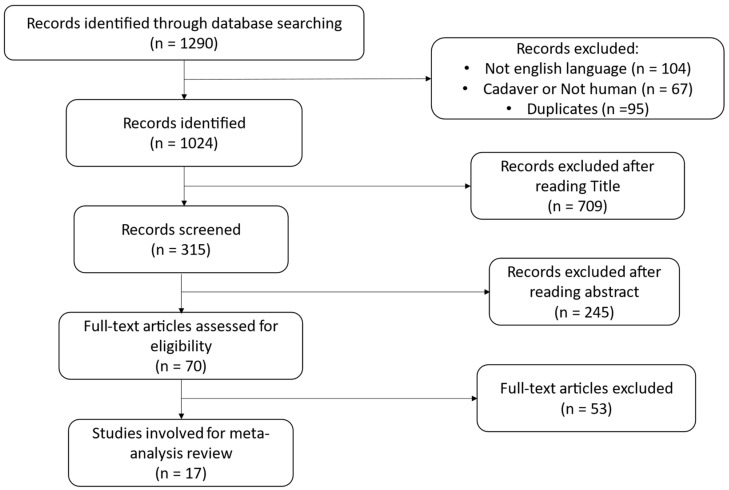
Study attrition diagram, outline of search process, and excluded studies in accordance with the Preferred Reporting Items for Systematic Reviews and Meta-Analysis (PRISMA) guidelines.

The literature search conducted through the PRISMA guidelines [31] is shown in Figure 1. The article has not been registered. In total, 1290 articles were identified through the initial search. A total of 104 articles were excluded because they were not written in the English language, 67 involved animals or cadavers, and 95 were duplicates; 1024 articles remained. After reading titles, 315 papers remained. This was further reduced to 70 articles after reading the abstracts. A further 53 articles were omitted as the full papers did not match the eligibility criteria. In total, 17 literature articles met the inclusion criteria and were eligible for the systematic review [13,16,30,32,33,34,35,36,37,38,39,40,41,42,43,44,45]. The process is shown in Figure 1.

Selected articles, demographic data and characteristics of the chosen studies are shown in Table 1. A total of 5061 patients underwent free-flap transfer to lower-extremity defects. The total mean age was 40.31 years, with a range of 1–93 years.

A total of 5133 free flaps were performed (see Table 2). Among the fasciocutaneous flaps, the ALT flap was the most utilized (*n* = 877, 17.12%), followed by the SCIP flap (*n* = 215, 4.20%) and parascapular flap (*n* = 201, 3.92%); among muscle flaps, the latissimus dorsi (LD) was the most used (*n* = 820, 16.01%), followed by the rectus abdominis (*n* = 586, 11.44%) and gracilis (*n* = 369, 7.20%); regarding bone flaps, the most frequently performed were the fibula flap (*n* = 18, 0.35%) and medial femoral condyle (*n* = 5, 0.10%).

Globally, 1396 (27.25%) fasciocutaneous flaps, 1850 (36.11%) muscle flaps and 23 (0.45%) bone flaps were performed.

The most common sites of the defect were the lower leg in 52.19% of cases (*n* = 2686), the foot in 23.90% of cases (*n* = 1230), and the ankle in 4.93% of cases (*n* = 254) (see Table 3).

The causes of the defects were related to traumas in 79.40% (*n* = 4055), tumors in 5.13% (*n* = 262), infections in 2.39% (*n* = 122), and diabetes in 2.33% (*n* = 119) (see Table 4).

Globally, 367 (7.78%) cases of total flap necrosis, 415 (9.15%) cases of partial flap necrosis, and 647 (13.79%) cases of return to the operating room for suspected microvascular compromise (see Table 1) were recorded.

Causes of return to the operating theater are outlined in Table 5. Arterial occlusion occurred in 109 cases (30.88%), venous thrombosis in 193 cases (54.68%), and hematoma in 38 cases (10.76%). Other unmentioned causes were found in 13 cases (3.68%).

## 4. Discussion

As described previously, lower limb reconstruction represents a challenge for plastic surgeons, and it is burdened by higher rates of complications than other anatomical regions [2,3,4,5,6,7,8]. Before performing any procedures, an accurate evaluation of the specific clinical case should take place.

In fact, beyond the localization and size of the defect and the type of missing tissue, the causes that produced it can be countless: trauma, oncological resection, chronic wound, bedsores, diabetes, infections, venous or lymphatic stasis, peripheral vascular disease, and nerve injury. In contrast to several other studies investigating lower limb free tissue transfer, this research has analyzed a heterogeneous patient cohort not limited only to traumatic mechanisms.

In our systematic review, most defects were localized in the lower leg (52.19%, *n* = 2686) and caused by trauma (79.40%, *n* = 4055).

An analysis of the surrounding tissues must also be performed, and an initial surgical debridement of any devitalized tissue is usually necessary. Thorough evaluation of the condition of underlying soft tissues, bones, tendons, ligaments and vessels must be carried out [46].

Another aspect is the recipient vessel availability in terms of the size, length and preservation of the three vascular axes, which are not always intact. Traditionally, anastomosis to the major axial vessels and an end-to-side anastomosis have been the gold standard [37,47]. Some authors have tried to improve flap survival rates by adding a second venous anastomosis, which has been shown to have a protective effect, reducing overall complication and flap failure rates [48].

Recently, Power et al. [13] demonstrated that using a major artery or a perforator as a recipient, the total and partial flap failure rates were equivalent to and comparable with the published literature [49,50]. Previous research has revealed no influence on flap outcomes related to either vessel selection [51] or anastomosis configuration (end-to-end versus end-to-side) [52,53].

Moreover, orthopedic intervention is sometimes required and a multidisciplinary team approach must be used [53].

The last issue faced is the choice of flap. Nowadays, reconstructive surgeons have numerous tools at their disposal, and careful planning is necessary to establish which flap would be the most appropriate to reconstruct a specific defect.

Traditionally, muscle flaps were used because of their capacity to fill dead space and because it was believed that their reliable vascular supply decreased microbial load and promoted bone union [14,15,16,17]. However, they were burdened by greater morbidity of the donor site. On the other hand, fasciocutaneous flaps avoid muscle sacrifice and may result in a better aesthetic contour with thin, pliable tissue (especially for the ankle, foot, heel, and sole) [18,19]. Many studies demonstrated the same reconstructive, functional and bone union outcomes between muscle and fasciocutaneous flaps [20,21,22,23,24], and the debate is still open. In our study, the most used fasciocutaneous flap was ALT (17.12%, *n* = 877), and the most used muscle flap was LD (16.01%, *n* = 820). The majority of flaps were muscle flaps, accounting for 36.11% (*n* = 1850) of flaps in comparison with fasciocutaneous/perforator flaps, which accounted for 27.25% (*n* = 1396) of flaps. These data are very interesting. In fact, despite the demonstrated substantial equivalence in terms of success and the lower donor site morbidity offered by fasciocutaneous flaps, large studies published in the last seven years still highlight a tendency to use muscle flaps more frequently.

Finally, the ultimate goal of such reconstructions is to restore the form, function and contour of the limb in question [54], considering that every specific case requires specific preoperative planning and a specific reconstruction.

The aim of our systematic review was to highlight the rate of total and partial flap failure and the rate of return to the operating room for suspected vascular compromise in lower limb reconstruction.

Based on 5061 patients and 5133 free flap reconstructions extracted from 17 articles in English that were published in the last 7 years (2017–2024), the present study provides a high level of evidence of the procedural outcome and safety in microsurgical reconstruction of defects in the lower extremities.

The analysis showed a total flap failure rate of 7.78% (*n* = 367), a partial flap failure of 9.15% (*n* = 415), and a return rate of 13.79% (*n* = 647).

In our paper, the most frequent cause of return to the operating theatre was venous thrombosis (54.68%, *n* = 193), followed by arterial occlusion (30.88%, *n* = 109) and hematoma (10.76%, *n* = 38). Not all of the studies analyzed mentioned the causes of return, and this represents a limitation. Nevertheless, it shows a general trend that could suggest the possibility of performing two venous anastomoses.

Hao Liu et al. [45]’s study showed the highest rate of total flap failure (13.11%); Carney et al. [37]’s study showed the lowest (3.13%).

The highest partial flap failure rate was reported by Lee et al. [16] (15.2%), and the lowest by Hao Liu et al. [45] (1.23%).

Heidekrueger et al. [40] referred the highest return rate (19%), and Carney et al. [37] the lowest (4.69%).

In Xiong et al. [49]’s meta-analysis published in 2016 and including papers from 2000 to 2014, a total flap loss rate of 6% and a partial flap loss rate of 6% were reported.

The complete failure of a free flap poses a significant challenge for the surgeon. In fact, a pedicled flap is often not available, and a simple skin graft is not the ideal choice. According to a study conducted by Koster et al. [55], following the complete failure of a free flap, a new free flap is performed in 69% of cases. The failure rate for the second free flap was 17%. Overall, 12% of cases experiencing failure of the first flap underwent amputation, while the same fate befell 50% of patients in whom the second flap also failed. In cases of partial necrosis of a free flap, however, a skin graft proved to be the most commonly performed option (50%) with a high success rate. Other possibilities include healing by secondary intention and the use of devices such as VAC therapy.

Compared with data of free-flap microsurgical reconstruction of other anatomical regions like breast reconstruction [55,56], rates in lower limb reconstruction are obviously higher and reflect an increased procedural risk due to the factors mentioned above.

Having said that, with respect to the complexity of microsurgical reconstruction of the lower extremities, the analyzed complication rates demonstrate the safety of the procedure.

The results are general but offer a wide view of what patients and surgeons could expect from a reconstruction of a lower limb defect with a free flap.

## 5. Limitations

This study is not without limitations. Only studies published over the last seven years (2017–2024) were included in order to give an idea of the recent trends. Moreover, the choice of the papers was conducted by two authors, which may have led to bias or articles being missed. The retrieved studies are all retrospective case series with a level of evidence of 4.

In four studies, the type of flap was not extractable, and in three studies, the site of the defect was not specified.

Overall limb salvage rates were not extracted, which may give more beneficial functional outcomes.

Many of the papers included in our study did not show data regarding the gender of the patients, tobacco use, or previous diseases like vasculopathies or diabetes.

A huge number of flaps have been analyzed, but 36.19% are still part of the category “other flaps”, therefore remaining unknown.

Further categories such as defect size, follow-up duration, time to soft-tissue coverage, donor/recipient vessel diameters, individual flap-type failure rates, reason for flap failure, and Gustilo–Anderson classification of injuries were beyond the scope of this study but would add further insight into flap selection and successful limb salvage.

Moreover, this systematic review was limited by the available published studies that summarize various techniques (performed by different surgeons in different centers), which are highly variable and not standardized. We decided to include the studies with the highest level of evidence and biggest number of patients possible; consequently, a high number of case reports were excluded.

The nature of such surgery also makes it difficult to standardize patient selection (comorbidities, localization, defect size, and etiology) and flap anatomy (defect size, number of perforators, length of pedicle, and quality of recipient vessels).

In the light of what has been said so far, the results are broad and general and may not account for individual factors that influence optimal flap choice. 

## 6. Conclusions

Microsurgical reconstruction of defects in the lower limb reconstruction could be considered safe and reliable.

This paper contributes to the plastic surgery literature by highlighting the latest international standards to be following in the field of lower limb reconstruction.

Arguably, flap selection should always be individualized to the defect location, size and aesthetic and functional demand of the patient, and accurate debridement should always be performed before reconstruction.

We believe that our systematic review, implemented with future research, could be a motivation to plastic surgeons to reach the international standards described in it.

## Figures and Tables

**Table 1:** Demographic data.

Study	No. of Patients	No. of Flaps	Age, Means	Range	No. Total Flap Failure	% Total Flap Failure	No. Partial Flap Failure	% Partial Flap Failure	No. Flap Take Back	% Flap Take Back
Martin J. Carney et al. (2020) [37]	128	128	47.43	/	4	3.13	4	3.13	6	4.69
Hollie A. Power et al. (2022) [13]	407	423	46.20	4–81	26	6.14	38	8.98	39	9.22
Z-Hye Lee et al. (2019) [16]	165	165	35.00	/	6	3.60	25	15.20	21	12.70
John T. Stranix et al. (2019) [41]	358	358	37.42	4–83	30	8.38	37	10.34	68	18.99
John T. Stranix et al. (2018) [30]	481	481	36.40	3–83	37	7.70	45	9.40	71	14.80
Nicholas Moellhoff et al. (2022) [33]	358	393	52.69	18–93	29	7.38	15	3.82	69	17.55
Z-Hye Lee et al. (2020) [35]	393	393	36.27	/	33	8.40	35	8.90	61	15.50
Cara Black et al. (2020) [36]	115	115	55.90	19.4–87.5	8	7.00	11	9.60	6	5.20
John T Stranix et al. (2020) [38]	373	373	42.22	3–83	20	5.40	29	7.80	29	7.80
Harrison Theile et al. (2022) [32]	234	234	/	/	9	3.80	13	5.50	22	9.40
Paul I. Heidekrueger et al. (2019) [40]	89	100	53.24	18–88	9	9.00	6	6.00	19	19.00
John T. Stranix et al. (2018) [42]	361	361	37.51	9–80	31	8.60	37	10.30	45	12.40
Hani I. Naga et al. (2021) [34]	173	173	47.45	/	10	5.78	18	10.40	14	8.09
John T. Stranix et al. (2018) [43]	362	362	37.00	/	29	8.00	40	11.00	44	12.20
Z-Hye Lee et al. (2019) [39]	410	410	36.22	/	34	8.29	35	8.54	61	14.88
Hao Liu et al. (2023) [45]	244	244	42.40	1–71	32	13.11	3	1.23	35	14.34
Joani M. Christensen et al. (2024) [44]	410	420	52.00	38–60	20	4.76	24	5.71	37	8.80
Total	5061	5133	40.31	1–93	367	7.78	415	9.15	647	13.79

**Table 2:** Types of flaps.

Flap Type	Total	%
Fasciocutaneous		
Parascapular	201	3.92%
Lateral arm	17	0.33%
Radial forearm	57	1.11%
DIEP	1	0.02%
Groin	27	0.53%
SCIP	215	4.20%
ALT	877	17.12%
AMT	1	0.02%
Muscle		0.00%
Deltoid	3	0.06%
Latissimus dorsi	820	16.01%
Serratus	25	0.49%
Rectus abdominis	586	11.44%
Rectus femoris	4	0.08%
Vastus lateralis	34	0.66%
TFL	9	0.18%
Gracilis	369	7.20%
Bone		0.00%
Medial femoral condyle	5	0.10%
Fibula	18	0.35%
Other (not specified)	1854	36.19%
Total	5123	

**Table 3:** Sites of defects.

Defect Location	Number of Flaps	%
Thigh	40	0.78%
Knee	67	1.30%
Lower leg	2686	52.19%
Ankle	254	4.93%
Foot	1230	23.90%
Toes	0	0.00%
Others (not specified)	870	16.90%
Total	5147	

**Table 4:** Causes of defects.

Cause of the Defect	Total	%
Trauma	4055	79.40%
Tumor	262	5.13%
PTS	75	1.47%
Infection	122	2.39%
Diabetes	119	2.33%
Radiation	7	0.14%
PVD	81	1.59%
Previous surgery	6	0.12%
Other (not specified)	380	7.44%
Total	5107	

**Table 5:** Causes of return to the operating theatre.

Study	No. of Flaps	No. Flap Take Back	% Flap Take Back	Causes of Take Back
Martin J. Carney et al. (2020) [37]	128	6	4.69	Arterial occlusion: 1 Venous thrombosis: 4 Others (not mentioned): 1
Hollie A. Power et al. (2022) [13]	423	39	9.22	Arterial occlusion: 25 Venous thrombosis: 37 Hematoma: 2
Z-Hye Lee et al. (2019) [16]	165	21	12.70	Not mentioned
John T. Stranix et al. (2019) [41]	358	68	18.99	Arterial occlusion: 17 Venous thrombosis: 27
John T. Stranix et al. (2018) [30]	481	71	14.80	Arterial occlusion: 22 Venous thrombosis: 35 Hematoma: 7 Others (not mentioned): 7
Nicholas Moellhoff et al. (2022) [33]	393	69	17.55	Arterial occlusion: 13 Venous thrombosis: 33 Hematoma: 23
Z-Hye Lee et al. (2020) [35]	393	61	15.50	Not mentioned
Cara Black et al. (2020) [36]	115	6	5.20	Not mentioned
John T Stranix et al. (2020) [38]	373	29	7.80	Not mentioned
Harrison Theile et al. (2022) [32]	234	22	9.40	Not mentioned
Paul I. Heidekrueger et al. (2019) [40]	100	19	19.00	Arterial occlusion: 4 Venous thrombosis: 9 Hematoma: 6
John T. Stranix et al. (2018) [42]	361	45	12.40	Arterial occlusion: 14 Venous thrombosis: 26 Others (not mentioned): 5
Hani I. Naga et al. (2021) [34]	173	14	8.09	Not mentioned
John T. Stranix et al. (2018) [43]	362	44	12.20	Not mentioned
Z-Hye Lee et al. (2019) [39]	410	61	14.88	Not mentioned
Hao Liu et al. (2023) [45]	244	35	14.34	Not mentioned
Joani M. Christensen et al. (2024) [44]	420	37	8.80	Arterial occlusion: 13 Venous thrombosis: 22
Total	5133	647	13.79	Arterial occlusion: 109 (30.88%) Venous thrombosis: 193 (54.68%) Hematoma: 38 (10.76%) Others (not mentioned): 13 (3.68%)
